# Characterization of HER2-Positive Murine Breast Cancer Models for Investigating HER2-Targeted Therapy and Immunotherapy

**DOI:** 10.3390/cancers18060997

**Published:** 2026-03-19

**Authors:** Yun Lu, Benjamin P. Lee, Abbigael V. Eli, Shannon E. Lynch, Ar Rafi Md Faisal, Jonathan Moye, Anna G. Sorace

**Affiliations:** 1 Department of Radiology, University of Alabama at Birmingham, Birmingham, AL 35294, USA; yunlu@uab.edu (Y.L.); bplee@uab.edu (B.P.L.); slynch97@uab.edu (S.E.L.); jmoye@uab.edu (J.M.); 2 Medical Scientist Training Program, University of Alabama at Birmingham, Birmingham, AL 35294, USA; 3 Graduate Biomedical Sciences, University of Alabama at Birmingham, Birmingham, AL 35294, USA; aveli@uab.edu (A.V.E.); afaisal@uab.edu (A.R.M.F.); 4 Department of Otolaryngology, University of Alabama at Birmingham, Birmingham, AL 35233, USA; 5 Department of Biomedical Engineering, University of Alabama at Birmingham, Birmingham, AL 35294, USA; 6 O’Neal Comprehensive Cancer Center, University of Alabama at Birmingham, Birmingham, AL 35233, USA

**Keywords:** breast cancer, HER2+ breast cancer, brain metastasis, trastuzumab, tucatinib, anti-PD-1, anti-CTLA-4, T-DM1, T-DXd

## Abstract

**Background/Objectives**: Human epidermal growth factor receptor 2 (HER2)-positive breast cancer is linked to poorer overall survival and a higher risk of brain metastases compared to HER2-negative breast cancer. Current preclinical studies lack robust HER2+ metastatic syngeneic mouse models for investigating targeted and immunomodulatory therapies. This study aims to develop effective HER2+ mouse models to investigate response dynamics to HER2-targeted therapy and immunotherapy. **Methods**: The human HER2 gene (WT or mutant p.A775_G776insYVMA, GFP-tagged at the C-terminus) was introduced into triple-negative breast cancer (TNBC) mouse mammary carcinoma cells with known metastatic potential (4T1 and EO771) via lentiviral transduction. HER2 expression and phosphorylation were analyzed using Western blotting and immunohistochemistry. Tumors were treated with HER2-targeted therapy (trastuzumab and tucatinib), immune checkpoint blockade (anti-PD-1 and anti-CTLA-4), and anti-HER2 antibody–drug conjugate (ADC) to evaluate treatment efficacy. Metastatic potential was assessed with brain fluorescence imaging. Statistical analysis included ANOVA and Kaplan–Meier tests. **Results**: Newly established lines demonstrated expression of HER2+, with HER2^YVMA^ lines showing higher phosphorylation than HER2^WT^ lines. Cells were tumorigenic, demonstrating in vivo tumor take rates at 100% for 4T1-HER2 and 15–30% for EO771-HER2. HER2 overexpression led to a 30% increase in spontaneous brain metastasis in the 4T1-HER2 models. Trastuzumab alone did not reduce primary tumor size but significantly reduced brain GFP signal by 17% ± 8% and 26% ± 7% in the 4T1-HER2^WT^ and 4T1-HER2^YVMA^ models, respectively. Combinational therapies with anti-HER2 therapy and immune checkpoint blockade effectively suppressed primary tumor growth and prolonged survival in EO771-HER2^YVMA^ model. T-Dxd, but not T-DM1, demonstrated partial treatment response in the EO771-HER2^WT^ model. **Conclusions**: HER2+ syngeneic tumor models were developed that spontaneously metastasize to the brain and demonstrate variable responses to immunotherapies and ADCs. These models are valuable for advancing molecular imaging modalities for HER2+ brain metastasis, studying blood–brain barrier penetration of HER2-targeted drugs, and exploring the combination of therapies, including immunotherapy.

## Simple Summary

Mouse models are a key tool for investigating new cancer treatments. There is a need for new models of human epidermal growth factor receptor 2 (HER2)-positive breast cancer to investigate HER2-targeted therapy and immunotherapy in primary and metastatic disease. We sought to develop new HER2+ models by expressing human HER2 in mouse mammary carcinoma (breast cancer) cell lines. We then used these models to investigate various treatment strategies, including HER2-targeted therapies, immunotherapies, and HER2-targeting antibody–drug conjugates. In models with low HER2 expression, treatment with a HER2-targeted antibody appeared to decrease the rate of brain metastases despite not reducing the initial tumor size. Further, combination HER2-targeted therapy and immunotherapy significantly prolonged survival compared to control. While antibody–drug conjugates reduced tumor size in human breast cancer cell line models, these therapies did not significantly impact tumor growth in our newly developed models. Taken together, these models provide new avenues for investigating HER2+ breast cancer therapies.

## 1. Introduction

Human epidermal growth factor receptor 2-positive (HER2+) breast cancer is characterized by aggressive tumor behavior and heightened metastatic potential, particularly to the central nervous system [1,2,3]. Despite advances in HER2-targeted therapies, brain metastases remain a common complication of HER2+ disease and are associated with significant neurological morbidity and poor clinical outcomes [2]. Current treatments, including monoclonal antibodies (e.g., trastuzumab, pertuzumab), antibody–drug conjugates (ADCs, e.g., trastuzumab emtansine (T-DM1), trastuzumab deruxtecan (T-DXd)), and tyrosine kinase inhibitors (TKIs, e.g., tucatinib), improve systemic control but exhibit limited efficacy against established brain metastases and often require adjunctive radiotherapy or surgery [4,5]. Survival remains poor due to heterogeneity of therapeutic response and development of therapeutic resistance, underscoring the need for novel strategies [6,7]. HER2-targeting ADCs like T-DXd have expanded in their clinical utility in recent years for the treatment of both primary and metastatic HER2+ disease as well as other cancers [8]. Intriguingly, T-DXd has also demonstrated some efficacy in treating brain metastases in HER2-low populations, a subset of breast cancer previously considered HER2-negative that now defines a portion of triple-negative and hormone receptor positive breast cancer [9,10,11,12,13]. While it remains unclear if HER2-low disease is associated with an increased rate of brain metastases or decreased overall survival, studies have identified varied HER2 expression over time as well as across primary and metastatic lesions, including increased HER2 expression in brain metastases [10,14,15,16]. Across these groups, however, durable responses continue to remain suboptimal, underscoring the need to elucidate how HER2 expression levels and molecular alterations impact metastatic potential and therapeutic response [14,17,18].

Despite therapeutic progress, preclinical models inadequately recapitulate the natural progression of HER2-driven metastasis, particularly to the brain [19,20]. Existing systems often lack fidelity to the blood–brain barrier and tumor microenvironment, hindering mechanistic studies of drug penetrance and resistance [20,21]. Current models also fail to replicate the heterogeneity of HER2 expression, including HER2-low tumors, and stromal interactions observed clinically [22,23], complicating ADC optimization. Similarly, investigations into emerging immunotherapies like anti-HER2 vaccines and immune checkpoint blockade (ICB) are limited by the absence of immunocompetent models that mirror metastatic HER2-low or HER2+ disease. While some murine HER2+ tumors exist, these express the murine ortholog of HER2 which is not targeted by current, FDA-approved human HER2-targeted therapies. These limitations hinder exploration of immune evasion mechanisms and evaluation of possible synergy of combination therapy. While some recent work has begun to address this by expressing HER2 in murine models, these studies predominantly focused on the use of HER2-targeted therapy and did not explore the addition of immunotherapy or antibody–drug conjugates [24,25]. Together, there remains a need for advanced platforms that integrate dynamic metastasis, immune crosstalk, and HER2 heterogeneity to bridge preclinical and clinical outcomes.

A key molecular driver of HER2+ oncogenesis is the in-frame insertion at residue G776 (p.A775_G776insYVMA, hereafter referred to as HER2^YVMA^) in exon 20, observed in lung and breast cancers [26,27]. This mutation induces potent autophosphorylation and transphosphorylation of kinase-dead EGFR/HER3, forming hyperactive heterodimers that disproportionately activate proliferative (MAPK/ERK) and antiapoptotic (PI3K/AKT) pathways compared to HER2 wild-type (HER2^WT^) [28,29]. In lung cancer, HER2^YVMA^ is enriched in brain metastases. This, combined with the increased rates of brain metastases in HER2+ breast cancer, may indicate conserved neurotropic potential for HER2 across tumor types [27]. However, its role in breast-to-brain metastasis remains poorly defined. While HER2^YVMA^ tumors exhibit resistance to EGFR TKIs (e.g., erlotinib), they retain sensitivity to HER2-targeted agents like trastuzumab and TKIs (lapatinib, tucatinib) [28]. The lack of immunocompetent models with endogenous HER2^YVMA^ mutations limits investigation of the interplay with the brain microenvironment or immune system, which are key determinants of response to ICBs, ADCs, and targeted therapies. Addressing these gaps may unveil mutation-specific therapeutic strategies for brain-metastatic disease.

To overcome these challenges, we engineered syngeneic TNBC models (4T1, EO771) with metastatic potential to stably express human HER2^WT^ or the exon 20 mutant HER2^YVMA^, C-terminally tagged with GFP for in vivo tracking. Lentiviral transduction achieved HER2 expression, with phosphorylation and signaling activity validated via Western blotting and immunohistochemistry. These immunocompetent, isogenic models recapitulate HER2 heterogeneity (HER2-low/mutant) and enable therapeutic testing with trastuzumab, tucatinib, ADCs, and ICBs (anti-PD-1, anti-CTLA-4). Longitudinal GFP imaging quantified brain metastasis, while combination therapies assessed vulnerabilities in HER2-driven neurotropism. By integrating HER2 mutation status with immune-modulating regimens, this platform addresses critical gaps in modeling natural metastasis and resistance mechanisms, offering insights into therapeutic synergies to combat HER2-low and HER2-mutant brain metastases.

## 2. Materials and Methods

### 2.1. Lentivirus Plasmid Cloning and Packaging

Plasmids encoding human HER2 wild-type (HER2^WT^; Addgene, Watertown, MA, USA, #16257) and the constitutively active mutation p.A775_G776insYVMA (HER2^YVMA^; Addgene, #40982) were obtained from Addgene. The HER2^WT^ and HER2^YVMA^ variant were PCR-amplified with the NEBNext High-Fidelity 2X PCR master mix (NEB, Ipswich, MA, USA, M0541S) in 50 μL total volume using the following primers: forward 5′-ACTGTCTAGAATGGAGCTGGCGGCCTTGT-3′ and reverse 5′-ACTGTCTAGATCACACTGGCACGTCCAGA-3′. Thermocycling was performed for 40 cycles, with denaturation at 98 °C for 10 s, annealing at 60 °C for 30 s, and amplification at 72 °C for four minutes. PCR products were resolved via 1% agarose gel electrophoresis (Thermo Fisher Scientific, Waltham, MA, USA, 17850) and visualized with SYBR Safe (Thermo Fisher Scientific, S33102) before being purified with the QIAquick PCR & Gel Cleanup Kit (QIAGEN, Hilden, Germany, #28506).

For lentiviral vector construction, both the pLenti-CMV-MCS-GFP-SV-puro plasmid (Addgene, #73582) and the PCR-amplified HER2^WT^ or HER2^YVMA^ insert were digested with XbaI (NEB, R0145L) and subsequently ligated using T4 DNA ligase (NEB, M0202S). The resulting plasmid maps are included in Supplemental Figure S1. Lentiviral particles were generated by co-transfecting the recombinant HER2 lentiviral plasmid along with the packaging plasmids pCMV-VSV-G (Addgene, #8454) and pCMV-dR8.2 dvpr (Addgene, #8455) into HEK293T cells using the calcium phosphate precipitation method.

### 2.2. Cell Culture and Generation of HER2-Expressing Cell Lines

Triple-negative breast cancer (TNBC) mouse mammary carcinoma cell lines 4T1 (ATCC, Manassas, VA, USA, #CRL-2539) and EO771 (ATCC, #CRL-3461) were cultured in Roswell Park Memorial Institute (RPMI) 1640 medium (Thermo Fisher Scientific, 11-875-119) supplemented with 10% fetal bovine serum (FBS) and 1% L-glutamine or Dulbecco’s Modified Eagle Medium (DMEM) (Fisher, Waltham, MA, USA, 31-053-036) supplemented with 10% FBS and 1% L-glutamine, respectively.

Parental 4T1 and EO771 cells were transduced with lentiviruses encoding GFP, HER2^WT^, or HER2^YVMA^. Selection was performed using puromycin at a concentration of 2 µg/mL for 4T1 cells and 5 µg/mL for EO771 cells. As GFP was fused to the C-terminal region of HER2, HER2-expressing cells were identified by GFP fluorescence.

### 2.3. Western Blot

Western blotting was performed as previously described. Briefly, cells were lysed on ice using RIPA buffer with a protease inhibitor (Roche Applied Science, Penzburg, Germany). Protein concentrations were measured using the Pierce™ BCA Protein Assay Kit (Thermo Fisher Scientific, 23225). For each cell line, 20 μg of protein was denatured in SDS and β-mercaptoethanol, boiled at 95 °C for 5 min, separated on a NuPAGE Bis-Tris gel, and transferred onto a PVDF membrane. Membranes were blocked with 10% dry milk in TBST and incubated overnight at 4 °C with primary antibody. Blots were washed and incubated with secondary antibody for 1 h at room temperature before being developed using the Amersham ECL Western Blot Detection System (GE Healthcare, Chicago, IL, USA). Signal visualization was performed using an SRX-101A Medical Film Processor (Konica Minolta, Tokyo, Japan). Antibodies: HRP-conjugated mouse anti-human β-actin (Cell Signaling Technology (CST), Danvers, MA, USA, 12262S), rabbit anti-human HER2/ErbB2 (Cell Signaling Technology, 2242S), rabbit anti-human Phospho-HER2/ErbB2 (Tyr1221/1222) (Cell Signaling Technology, 2243S), and HRP-conjugated goat anti-rabbit IgG (Cell Signaling Technology, 7074S).

### 2.4. In Vivo Tumor Models

All animal experiments were approved by the Institutional Animal Care and Use Committee (IACUC) at the University of Alabama at Birmingham. All animals were housed in UAB Animal Resource Program (ARP) facilities at 73 °F ± 5°F and 50% ± 20% humidity with a 12 h day/night cycle, and every cage included an EnviroPak for enrichment. For 4T1-HER2 models, 2 × 10^5^ cells were orthotopically injected into the third mammary fat pad of 7-week-old Balb/c mice (Charles River Laboratories, Wilmington, MA, USA). Due to the low take rate of EO771-HER2 tumors, established tumors were excised and tumor pieces were transplanted into a new cohort of mice as described previously [30,31,32]. Briefly, C57BL/6 mice were injected with 5 × 10^5^ EO771-HER2 cells. Animals with established tumors were humanely euthanized via sustained isoflurane exposure (5% isoflurane for 5 min) followed by cervical dislocation. Tumors were isolated and cut into 0.5 × 0.5 mm pieces. Tumor pieces (which contain both tumor cells and stromal tissue) were subcutaneously implanted into the third mammary fat pad of 7-week-old C57BL/6 mice. Tumors were measured with calipers, and tumor volumes were calculated with the formula V = (4π/3) × (shortest diameter/2) × (shortest diameter/2) × (longest diameter/2). When the tumors reached 75–300 mm^3^, mice were entered into the study and randomized for treatments. Randomization was performed regardless of cage to avoid cage differences as a confounding variable. Tumor volume > 2000 mm^3^ or low body condition following UAB IACUC guidelines were used as endpoints. Euthanasia was performed humanely via sustained isoflurane exposure (5% isoflurane for 5 min) followed by cervical dislocation.

### 2.5. Treatment Timing and Dosing

To assess response to trastuzumab monotherapy, mice received trastuzumab (15 mg/kg, intravenously) or saline every three days for a total of five doses (n = 5 per group). One saline mouse was excluded due to a treatment error. For combination anti-HER2 ± immunotherapy experiments, mice were divided into four groups: saline control, anti-HER2 therapy (trastuzumab and tucatinib), ICB (αPD-1 and αCTLA-4), and the combination of anti-HER2 and ICB (n = 7 per group for long-term study; n = 4 per group for treatment day 5 takedown). Flow cytometry data from one animal in the ICB group had low counts which prevented analysis, and it was excluded. The anti-HER2 therapy group received trastuzumab (10 mg/kg, intravenously) every three days starting from treatment day 0 for a total of three doses, along with tucatinib (50 mg/kg, orally) administered daily for seven days from day 0. The ICB group received αPD-1 (Bio X Cell, Lebanon, NH, USA, BE0146) and αCTLA-4 (Bio X Cell, BE0164) at a dose of 200 µg per mouse, intravenously, every three days from treatment day 1 for a total of three doses. The combination group received both anti-HER2 and ICB treatments following the same dosing schedules. For evaluation of response to HER2 targeting ADCs, saline, T-Dxd (MedChemExpress, Monmouth Junction, NJ, USA, HY-138298), or T-DM1 (MedChemExpress, HY-P9921A) were administered 10 mg/kg via tail vein injection on treatment days 0 and 7 (n = 3 animals per group due to high drug cost). Researchers were not blinded to the conditions.

### 2.6. Flow Cytometry

Tumor samples were harvested on day 5 post-treatment and prepared for flow cytometry. Tumor tissues were minced and enzymatically digested with collagenase IV (Thermo Fisher Scientific, 17-104-019) at 37 °C for 30 min. The resulting cell suspension was filtered through a 40 µm strainer (Thermo Fisher Scientific, 08-771-1) to obtain single cells. Red blood cells were lysed using an ammonium chloride solution (Thermo Fisher Scientific, 00-4333-57), followed by washing with FACS buffer. Tumor cells were resuspended at a concentration of 500,000 cells/100 µL, and cell surface antigens were stained with fluorophore-conjugated antibodies: NIR viability dye (Thermo Fisher Scientific, L34975), CD45-BV510 (BD, 563891), CD3-APC (eBioscience, 17-0031-82), CD4-FITC (Thermo Fisher Scientific, 11-0041-82), CD8-PerCP-Cy5.5 (Thermo Fisher Scientific, 45-0081-82), Ly6C-eFluor 450 (Thermo Fisher Scientific, 48-5932-82), and F4/80-SB645 (Thermo Fisher Scientific, 64-4801-82). Data acquisition and analysis were performed as previously described [33].

### 2.7. Immunohistochemistry (IHC) and Immunofluorescence (IF)

IHC and IF were performed as previously described to evaluate HER2 expression and localization in vitro and in tumor tissues [32]. Briefly, for IF, cells grown in 96-well plates were fixed with 10% formalin for 30 min at room temperature and blocked with 1% BSA in PBS for 1 h. Cells were incubated overnight at 4 °C with primary rabbit anti-human HER2 antibody (Cell Signaling Technology, 2242S, 1:100 dilution), followed by incubation with Cy5-conjugated goat anti-rabbit secondary antibody (Jackson ImmunoResearch, West Grove, PA, USA, 711-175-152, 1:200) for 1 h at room temperature in the dark. Cells were stained with 1 mg/mL DAPI (Thermo Fisher Scientific, PI62247) in PBS for 5 min, then washed twice with PBS.

For IHC, formalin-fixed, paraffin-embedded tumor sections (5 μm) were deparaffinized, rehydrated, and subjected to antigen retrieval in citrate buffer (pH 6.0) at 90 °C for 10 min. Endogenous peroxidase activity was quenched with 3% hydrogen peroxide. Then, slides were cultured with 3% H_2_O_2_ for 10 min, blocking buffer (1% BSA in PBS with 0.05% Triton 100) for 30 min at room temperature, primary antibody (rabbit anti-human HER2 (CST, 2242S, 1:300)) overnight at 4 °C, and secondary antibody (anti-Rabbit-IgG, GTX83399, GeneTex, Irvine, CA, USA) for 30 min at room temperature. Brown staining was developed with DAB substrate kit (Vector, SK-4105) for 30 s, followed by hematoxylin counterstaining. Both IF and IHC images were acquired with an EVOS M7000 Imaging System (Thermo Fisher Scientific) under a 20× lens. QuPath software (v.0.5.1, Edinburg, UK) was used to threshold and quantify positive cells [34].

### 2.8. GFP Brain Imaging

To assess spontaneous brain metastases, mice were euthanized on day 33 after tumor cell injection, and whole brains were harvested and rinsed in PBS. Brains were imaged ex vivo with the skull removed using an IVIS system (Revvity, Hopkinton, MA, USA) with excitation at 480 nm and emission at 520 nm to capture GFP signal. Imaging settings (1-s exposure time, binning factor of 4, and a 12.5 cm field of view) were consistently applied across all samples to ensure comparability. GFP fluorescence was quantified using Living Image Software (v4.8.2, Revvity, USA). One animal was considered a significant outlier compared to the remaining animals, regardless of group, following a Grubbs outlier test (α = 0.01) and was excluded from quantitative analysis.

### 2.9. Statistical Analysis

All statistical analyses were performed using GraphPad Prism version 10.4.1. Data are presented as mean ± standard deviation (SD). For comparisons involving more than two groups, one-way or two-way analysis of variance (ANOVA) followed by Tukey’s or Sidak’s multiple comparisons tests was applied, as appropriate. Kaplan–Meier survival curves were analyzed using the log-rank (Mantel–Cox) test. For tumor volume analysis, a repeated measures ANOVA was performed between groups. Statistical assumptions were tested before performing analysis. *p*-values less than 0.05 were considered statistically significant. All experiments were performed with at least three biological replicates.

## 3. Results

### 3.1. Establishment of HER2 Overexpression (OE) and Activation Mouse Models

**Figure 1:**
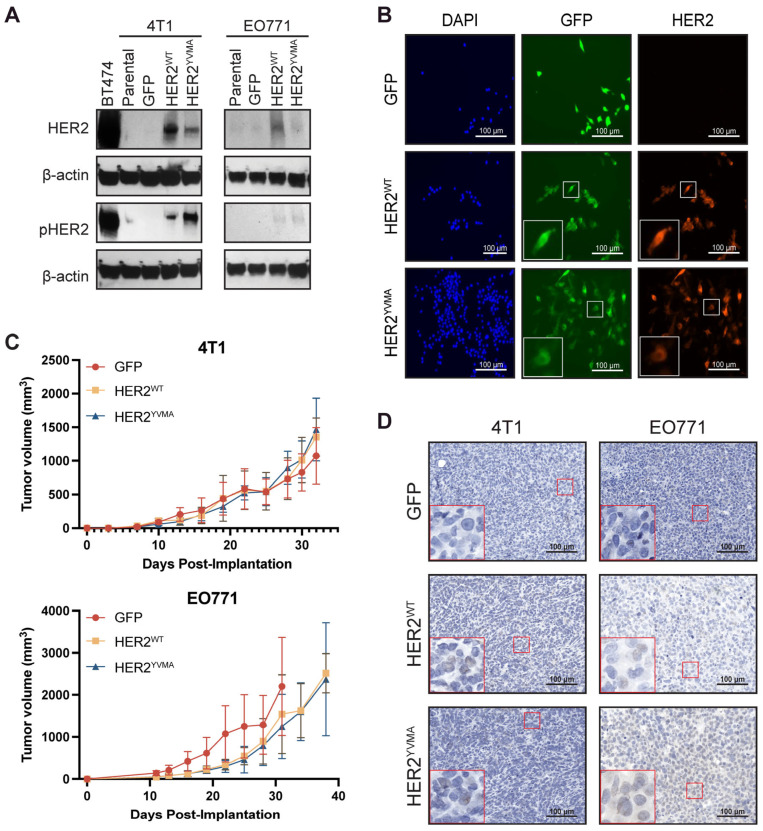
Validation of HER2 expression in the newly established HER2-positive mouse tumors. (**A**) Western blotting analysis of HER2 and phosphor-HER2 in transduced 4T1 and EO771 cell lines. BT474 included as HER2+ control. (**B**) Representative immunofluorescence staining of HER2 expression in transduced EO771 cells. (**C**) Tumor volume curve of 4T1 (**top**) and EO771 (**bottom**) HER2-OE lines (n = 4–5 per group). Data is mean ± SD. (**D**) Representative immunohistochemistry staining of HER2 expression on tumor slices. The uncropped blots are included in Supplementary File S1.

Lentiviral transduction successfully established human HER2-OE 4T1 and EO771 murine tumor models, validated via molecular assays (Figure 1). Western blotting confirmed HER2 expression in HER2^WT^ and HER2^YVMA^ lines at intermediate levels between high-HER2 BT474 and moderate-HER2 MDA-MB-361 human controls, with HER2^YVMA^ exhibiting elevated phosphorylation (activation) compared to HER2^WT^ (Figure 1A, controls in Supplementary Figure S2A). Immunofluorescence confirmed HER2 surface localization in transduced cells (Figure 1B, Supplementary Figure S2B). Tumor take rates were high (100%) for 4T1-HER2 models but low-moderate (15–30%) for EO771-HER2 models. Transplantation of established EO771-HER2 tumor pieces into new mice, as previously reported in other models [30,31,32], led to a 100% take rate. HER2 overexpression did not alter tumor growth rates in the 4T1 models and demonstrated a non-significant reduction in growth rate in the EO771-HER2 models (Figure 1C, raw data in Supplementary Table S1). Immunohistochemistry of in vivo tumors confirmed HER2 expression across the HER2+ models (Figure 1D). 4T1-HER2^WT^ and 4T1-HER2^YVMA^ models had 7.97% ± 4.14% and 28.71% ± 18.96% positive cells, respectively, compared to 0.72% ± 0.29% for the 4T1-GFP tumors. Similarly, the EO771-HER2 tumors had high expression compared to EO771-GFP, with EO771-HER2^WT^ and EO771-HER2^YVMA^ models having 28.21% ± 10.75% and 66.27% ± 16.27% positive cells, respectively, compared to 3.42% ± 2.03% for EO771-GFP. Together, this data supports the utility of these models for studying HER2-driven biology.

### 3.2. Trastuzumab Did Not Suppress Primary Tumor Growth but Reduced Metastatic Potential

**Figure 2:**
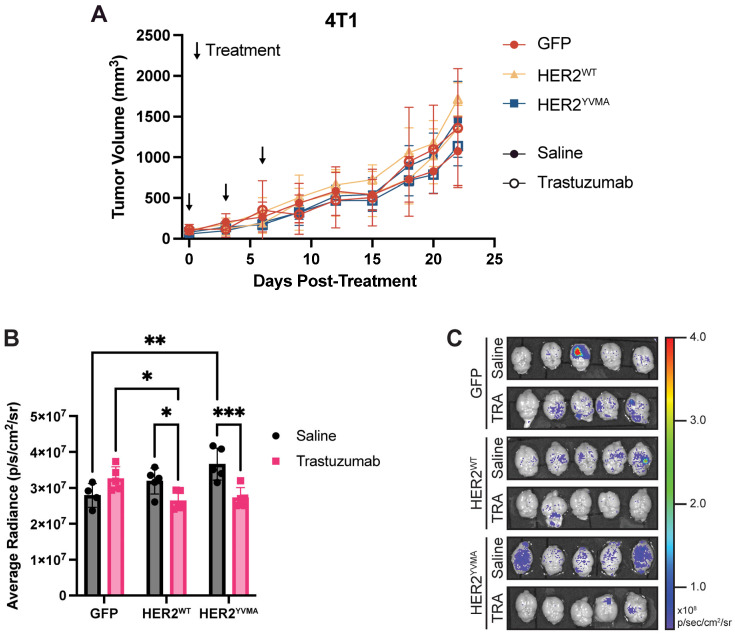
Trastuzumab alone was not able to reduce primary tumor size but decreased tumor brain metastasis in 4T1-HER2 models. (**A**) Tumor volume curve of 4T1-GFP, -HER2^WT^, and -HER2^YVMA^ tumors treated with either saline or trastuzumab (15 mg/kg, IV). Filled shapes represent saline-treated groups; open shapes represent trastuzumab-treated groups. No statistically significant difference was observed between groups (repeated measures ANOVA *p* = 0.56). (**B**) Plot of average radiance from GFP fluorescence in the brains of 4T1 tumor-bearing mice. Symbols represent individual mice. (**C**) Fluorescence images of the brains of 4T1 tumor-bearing mice. TRA, trastuzumab. Data is shown as mean ± SD. Two-way ANOVA with Tukey’s HSD test was used in (**B**). *, *p* < 0.05; **, *p* < 0.01; ***, *p* < 0.001.

Trastuzumab monotherapy demonstrated tissue-specific efficacy in HER2+ models (Figure 2). No significant changes in primary tumor volume were observed in 4T1-HER2^WT^, 4T1-HER2^YVMA^, or 4T1-GFP tumors treated with trastuzumab compared to saline controls (Figure 2A). Similarly, trastuzumab treatment did not reduce primary tumor growth in the EO771 GFP or HER2-OE models (Supplemental Figure S3). HER2^YVMA^ overexpression in 4T1 models led to a nearly significant increase in the frequency of spontaneous brain metastasis, with 1/5 HER2-negative mice developing metastasis compared to 8/10 across the HER2-expressing groups (Fisher’s Exact Test *p* = 0.0889). Quantitatively, HER2^YVMA^ tumors exhibited a 31% increase in brain GFP signal compared to HER2-negative controls (Figure 2B). Treatment with trastuzumab suppressed brain metastases in both the 4T1-HER2^WT^ and 4T1-HER2^YVMA^ cohorts, with a 17% ± 8% and 26% ± 7% decrease in GFP signal intensity, respectively (Figure 2B,C). No brain metastases were detected across the EO771 HER2-OE groups. These findings highlight trastuzumab’s potential role in suppressing HER2-driven metastasis, despite limited activity against primary tumors in both HER2 overexpressing 4T1 and EO771 models.

### 3.3. Combination of Trastuzumab Plus ICB Has Therapeutic Effects in Primary Tumors

**Figure 3:**
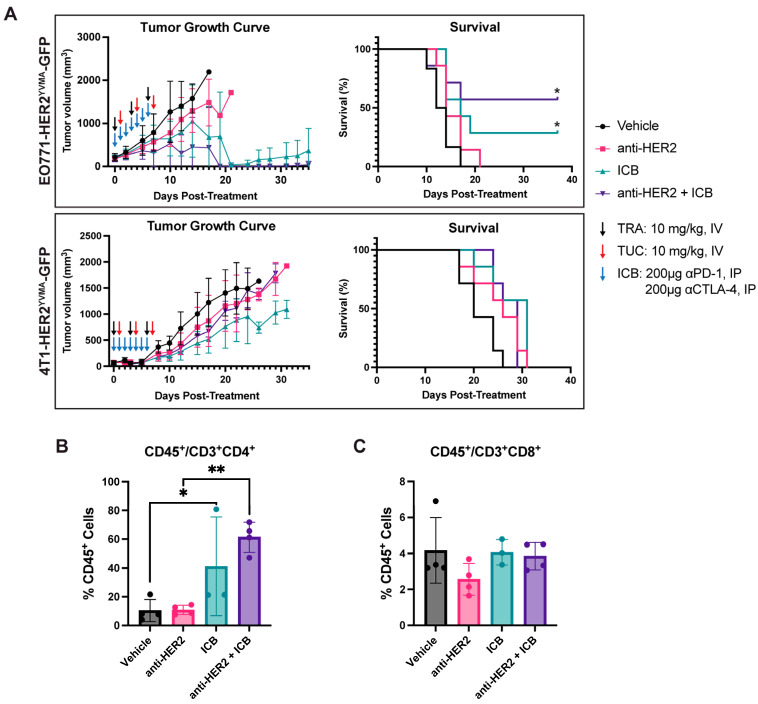
HER2-targeted therapy synergizes with immunotherapy in HER2-overexpressing (OE) tumors. (**A**) Tumor volume (**left**) and survival (**right**) curves for EO771-HER2^YVMA^ and 4T1-HER2^YVMA^ models treated with saline, anti-HER2 agents (TRA + TUC), ICB (αPD-1 and αCTLA-4), or combination therapy (anti-HER2 + ICB) as indicated by arrows. (**B**,**C**) Flow cytometry quantification of CD4+ (**B**) and CD8+ (**C**) T cells in EO771-HER2^YVMA^ tumors on day 5. Mantel–Cox logrank test was used for survival analysis in (**A**). Two-way ANOVA with Bonferroni correction for multiple comparisons was used for (**B**,**C**). All data are shown as mean ± SD; * *p* < 0.05; ** *p* < 0.01.

Combination anti-HER2 therapy (trastuzumab [TRA] + tucatinib [TUC]) with ICB (αPD-1 and αCTLA-4) suppressed primary tumor growth and significantly prolonged survival in the EO771-HER2^YVMA^ model compared to saline treatment (Figure 3A and Supplemental Figure S4A). However, in the 4T1-HER2^YVMA^ model, the combination therapy did not alter tumor volume compared to saline controls and survival remained unchanged (Figure 3A and Supplemental Figure S3B). Flow cytometry analysis on day 5 after treatment revealed a pronounced increase in tumor-infiltrating CD4+ T cells in the EO771-HER2^YVMA^ tumors following both ICB and combination anti-HER2 + ICB therapy (Figure 3B). Interestingly, no changes were observed in CD4+ T cells in the 4T1-HER2^YVMA^ tumors, mirroring the differences in therapeutic efficacy in these models (Supplemental Figure S4B). No change was observed in CD8+ T cells in either model (Figure 3C and Supplemental Figure S4C). These results underscore the combinatorial potential of HER2-targeted and immunotherapies in immunologically responsive HER2+ models, while highlighting context-dependent limitations in aggressive, poorly immunogenic settings like 4T1-HER2^YVMA^.

### 3.4. Varied Response to Anti-HER2 Antibody–Drug Conjugates (ADCs) in HER2-OE Models

**Figure 4:**
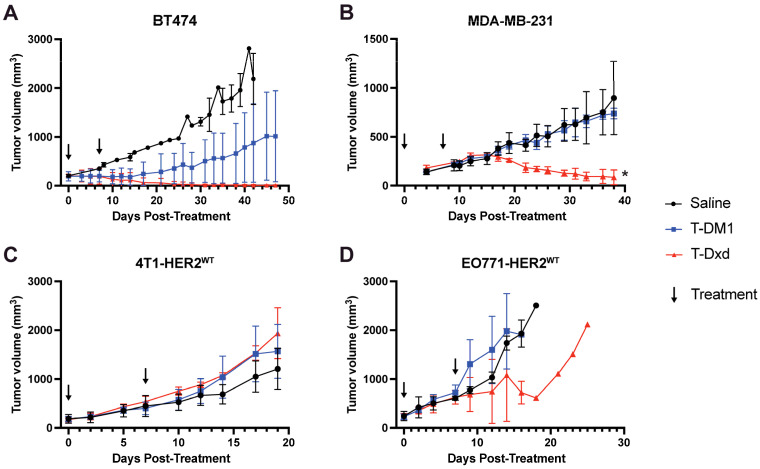
HER2-overexpressing models show differential responses to T-DM1 and T-Dxd. Tumor volume curves for BT474 (human HER2+) (**A**), HER2− MDA-MB-231 (human TNBC) (**B**), 4T1-HER2^WT^ (**C**), and EO771-HER2^WT^ (**D**) treated with saline (black line), T-DM1 (blue line), or T-Dxd (red line). n = 3 per group. * *p* < 0.05.

Anti-HER2 ADCs exhibited model-dependent efficacy (Figure 4). In HER2+ BT474 tumors, T-Dxd induced complete regression in all tumors, though one mouse required a soft diet due to weight loss. T-DM1 also suppressed growth in this model without adverse effects, though it did not lead to complete regression (Figure 4A and Supplemental Figure S5A). For HER2– MDA-MB-231 tumors, T-Dxd significantly reduced tumor volume, whereas T-DM1 showed no efficacy; both regimens were well-tolerated (Figure 4B and Supplemental Figure S5B). For our newly generated HER2-OE models, neither ADC impacted 4T1-HER2^WT^ tumors or mice body weight in our small cohort (Figure 4C and Supplemental Figure S4C). In EO771-HER2^WT^ models, T-Dxd reduced tumor volume in one mouse with some toxicity across all treated animals, while T-DM1 showed no efficacy (Figure 4D and Supplemental Figure S5D). Despite small sample sizes, these data highlight T-Dxd’s broader activity across HER2+/− models and potentially limited ADC efficacy in immunocompetent HER2+ settings (4T1/EO771) at tested doses and sample sizes.

## 4. Discussion

The development of immunocompetent HER2+ syngeneic models described in this study addresses a major gap in preclinical research on HER2-driven breast cancer brain metastasis and the role of combination therapies. Our findings confirm that lentiviral overexpression of human HER2^WT^ and the activating HER2^YVMA^ mutant in 4T1 and EO771 murine TNBC cells yields stable, surface-expressing HER2+ tumors with distinct phosphorylation and metastatic phenotypes. Importantly, HER2^YVMA^ appeared to increase spontaneous brain metastasis in vivo, consistent with known observations of HER2+ breast cancer in clinical contexts. These models uniquely enable dynamic tracking of brain dissemination using GFP imaging and provide a rare platform for mechanistic interrogation of HER2-driven metastatic progression and targeted or immune-modulating therapies in an immunocompetent setting.

Therapeutically, our data reveal important tissue-specific distinctions. While trastuzumab monotherapy had minimal impact on primary tumor growth, it significantly reduced brain metastases in the 4T1-HER2^YVMA^ model, suggesting targeting of metastatic potential. This finding mirrors clinical observations where trastuzumab may reduce extracranial metastatic burden [35,36]. The EO771 model, although HER2+, failed to develop overt brain metastases under the current protocol. Recent work has also identified distinct molecular subtypes within HER2+ breast cancer that can influence optimization and personalization of therapy [37]. With the growing landscape of therapeutic strategies for breast cancer, including for HER2+ breast cancer, these models offer an opportunity to probe new therapies and evaluate the efficacy and impact on the tumor microenvironment from combination therapies.

As the role of immunotherapy has continued to grow in breast cancer, there have been many clinical trials testing out the combination of HER2-targeting agents and immunotherapy (e.g., NCT04681287, NCT03032107). The combination of anti-HER2 agents (trastuzumab and tucatinib) with immune checkpoint blockade (anti-PD-1/CTLA-4) yielded strong therapeutic effects in the EO771-HER2^YVMA^ model, leading to suppressed tumor growth, enhanced immune infiltration, and prolonged survival. These results align with emerging clinical strategies combining HER2-targeted and immunotherapies and adds to the growing literature on the development of models to evaluate combination therapies [38,39,40,41]. Additionally, the use of two HER2-targeting agents, trastuzumab and tucatinib, mirrors approaches being investigated clinically, with the HER2Climb clinical trial demonstrating the effectiveness of this combination [42,43]. In contrast to the EO771-HER2^YVMA^ model, the 4T1-HER2^YVMA^ model showed limited response, consistent with prior work demonstrating low immunogenicity in parental 4T1 cells [31,44,45]. This work highlights how the tumor microenvironment and its intrinsic heterogeneity are critical determinants of ICB responsiveness. Therefore, this model pair may serve as a valuable tool to dissect immune resistance and identify predictive biomarkers for combination therapy efficacy and provide avenues for investigating the impact of combination therapies on downstream HER2 signaling. As PD-1 inhibitors are clinically used in breast cancer, this would provide a straightforward route to feed this method into clinical investigations.

ADCs have become an integral component of the treatment of metastatic HER2+ breast cancer. Results from the ADC experiments further underscore model-specific therapeutic outcomes. T-Dxd demonstrated broader activity across HER2+ and HER2− models, consistent with its bystander killing effect and clinical efficacy in HER2-low and HER2-mutant tumors [9,11]. However, T-DM1 only slowed tumor growth in the high-HER2+ BT474 model. These findings, paired with the lack of response in the 4T1-HER2 model and the partial response in EO771-HER2, suggest that HER2 surface levels alone are not sufficient for ADC efficacy in immunocompetent hosts, possibly due to altered drug penetration, immune-mediated clearance, or downstream signaling escape. Future studies should explore dose optimization, the role of stromal remodeling to enhance ADC delivery and function, and the combination of ADCs with immunotherapy. The role of combining ADCs plus immunotherapy could provide a three-pronged approach (targeted, cytotoxic, and immunotherapy) to treat more heterogeneous disease.

Despite the strengths of our HER2+ syngeneic models, several limitations warrant consideration. First, the variability in tumor take rate, particularly in the EO771-HER2 line, may introduce experimental inconsistency and complicate large-scale therapeutic screening. While tumor chunk transplantation improved take rates, this method may introduce stromal heterogeneity or alter immune dynamics compared to direct implantation, potentially limiting the relevance of this model. Second, while GFP-tagging facilitated non-invasive tracking of brain metastases, GFP fluorescence may underestimate micrometastatic burden or misrepresent deep parenchymal lesions due to signal attenuation. Other fluorophores or imaging modalities may increase detection of smaller or deep metastases and should be explored in future work. Additionally, the lack of overt brain metastases in EO771 models may reflect strain- or cell line-specific barriers to neuroinvasion, limiting its utility for studying CNS dissemination. Third, although we used standard dosing regimens for trastuzumab, tucatinib, and ADCs, differences in pharmacokinetics, immune engagement, and blood–brain barrier penetrance between mice and humans may alter the strategies needed for translational relevance. Future work incorporating alternative dosing or dose optimization strategies may provide further insight into response mechanisms. Finally, our study focused on short-term immune profiling predominantly focused on T cell populations as well as small survival cohorts for ADC studies due to drug cost constraints. The limited sample sizes tested in the ADC studies could limit detection of small numbers of responders in ADC studies. Future investigations incorporating longitudinal immune dynamics, including in myeloid populations, multi-omics profiling, and patient-derived variants would provide deeper mechanistic insights.

## 5. Conclusions

In conclusion, these newly established HER2+ syngeneic models, particularly those incorporating HER2 mutations, reproduce key clinical features of HER2-driven breast cancer metastasis, including brain tropism and heterogeneity in therapeutic response. These results provide key data on combination therapy response kinetics and offer an immunocompetent platform for testing HER2-targeted agents, ADCs, and immunotherapies in the context of spontaneous metastasis and blood–brain barrier constraints. These models will be critical for guiding the next generation of combination strategies for HER2+ and HER2-low breast cancer patients at risk for brain metastasis.

## Data Availability

The original contributions presented in this study are included in the article/Supplementary Material. Further inquiries can be directed to the corresponding author.

## Supplementary Materials

The following supporting information can be downloaded at https://www.mdpi.com/article/10.3390/cancers18060997/s1, Figure S1: Plasmid maps of HER2^WT^-GFP and HER2^YVMA^-GFP; Figure S2: Validation of HER2 overexpression cell lines; Figure S3: Trastuzumab alone did not reduce primary tumor size in EO771-HER2 models; Figure S4: HER2-targeted therapy and ICB combined in HER2-OE models; Figure S5: T-DM1 and T-DXd are well tolerated in breast cancer models; Supplementary Table S1: Figure Data; Supplementary File S1: Uncropped western blot images.

## Abbreviations

The following abbreviations are used in this manuscript:

ADC	Antibody–drug conjugate
CNS	Central nervous system
GFP	Green fluorescent protein
HER2	Human epidermal growth factor receptor 2
ICB	Immune checkpoint blockade
IF	Immunofluorescence
IHC	Immunohistochemistry
OE	Overexpression
T-DM1	Trastuzumab emtansine
T-DXd	Trastuzumab deruxtecan
TNBC	Triple-negative breast cancer
TRA	Trastuzumab
TUC	Tucatinib
WT	Wild-type
