# Supplementary material for: Characterization of HER2-Positive Murine Breast Cancer Models for Investigating HER2-Targeted Therapy and Immunotherapy
Source: Cancers (Basel). 2026 Mar 19;18(6):997. doi: 10.3390/cancers18060997 (PMC13024896; doi:10.3390/cancers18060997)
Supplement: Supplementary file 1 [file cancers-18-00997-s001.zip › supp_figures.pdf]

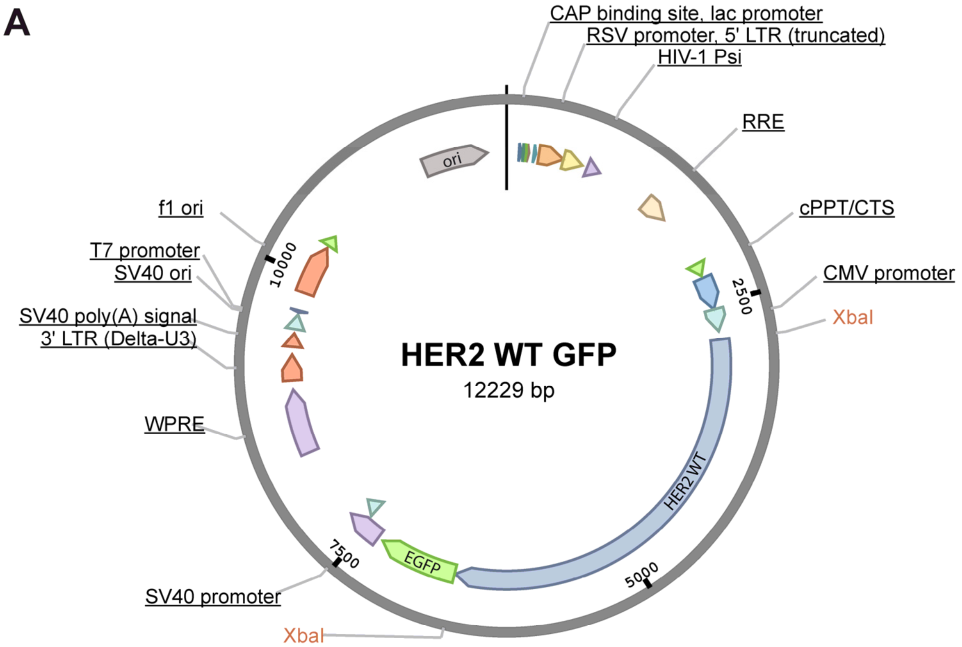

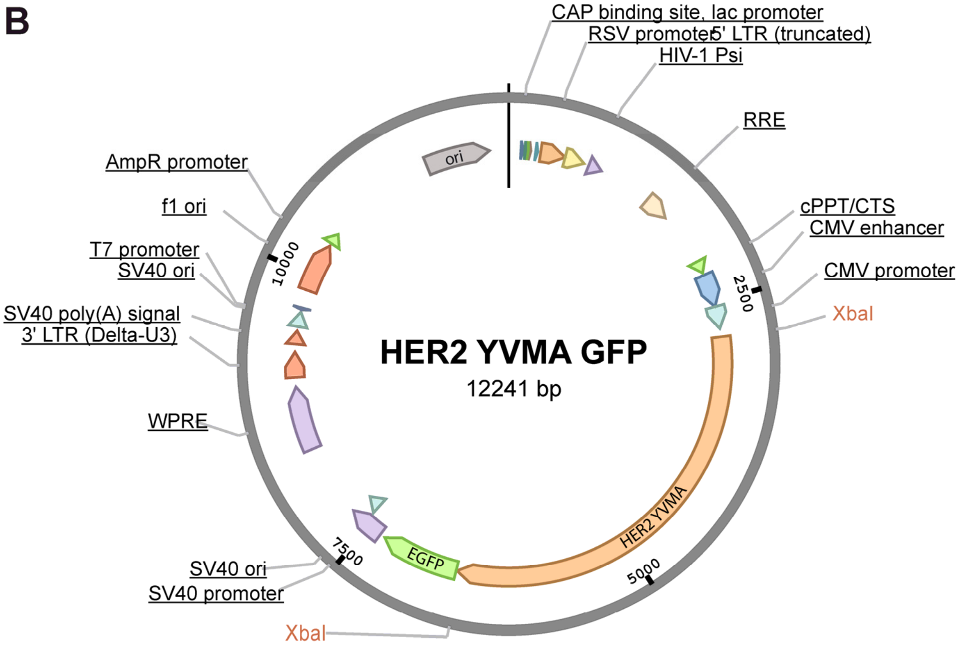

Supplemental Figure S1. Plasmid maps of HER2^{WT}-GFP and HER2^{YVMA}-GFP. (**A**) Plasmid map of HER2^{WT}-GFP plasmid. (**B**) Plasmid map of HER2^{YVMA}-GFP plasmid.

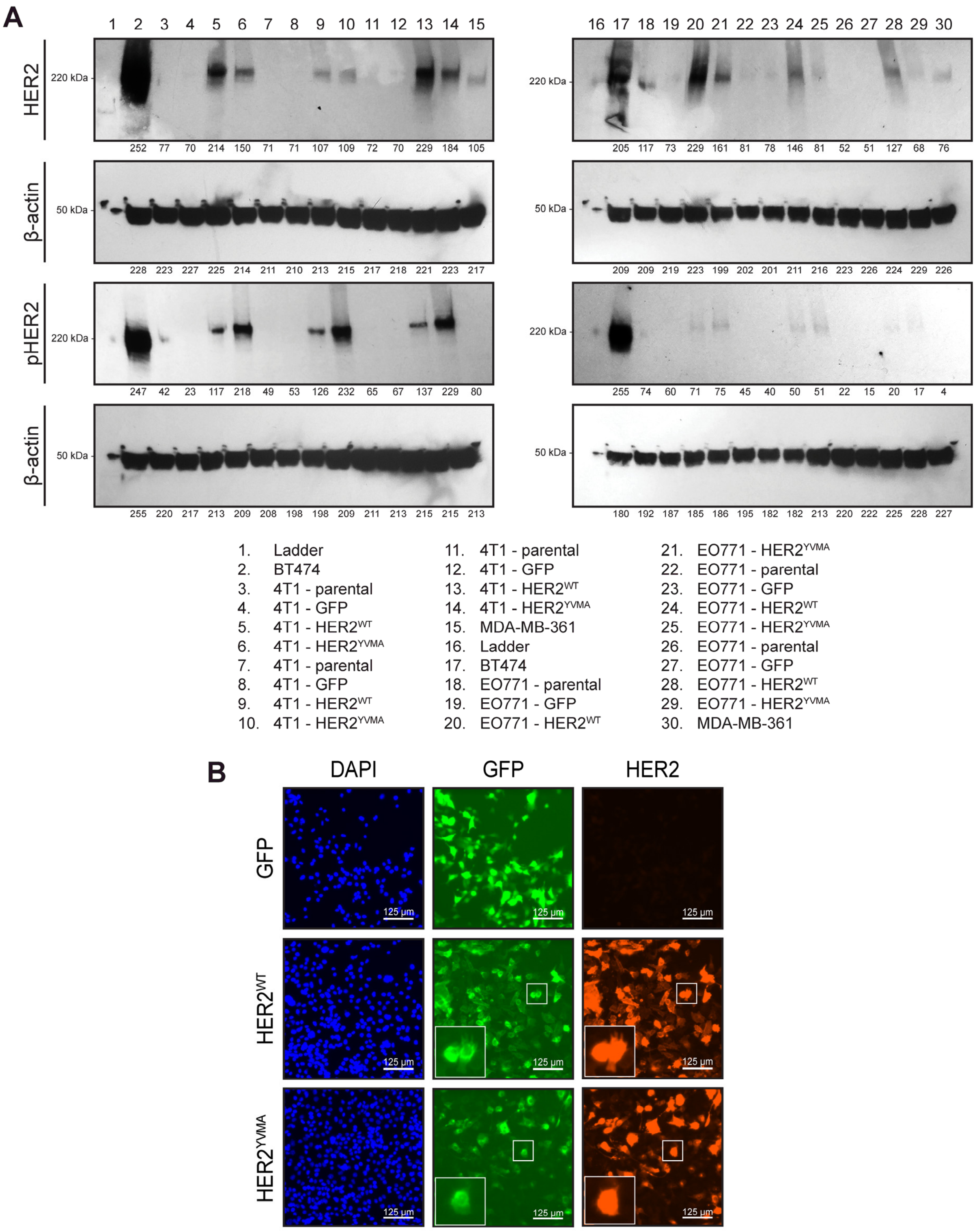

Supplemental Figure S2. Validation of HER2 overexpression cell lines. (**A**) Full western blot images of HER2 and p-HER2 in 4T1 and EO771 HER2-OE lines with three biological replicates. Densitometry values for each lane are indicated below each image. (**B**) Immunofluorescence staining of HER2 expression in 4T1-HER2 OE models.

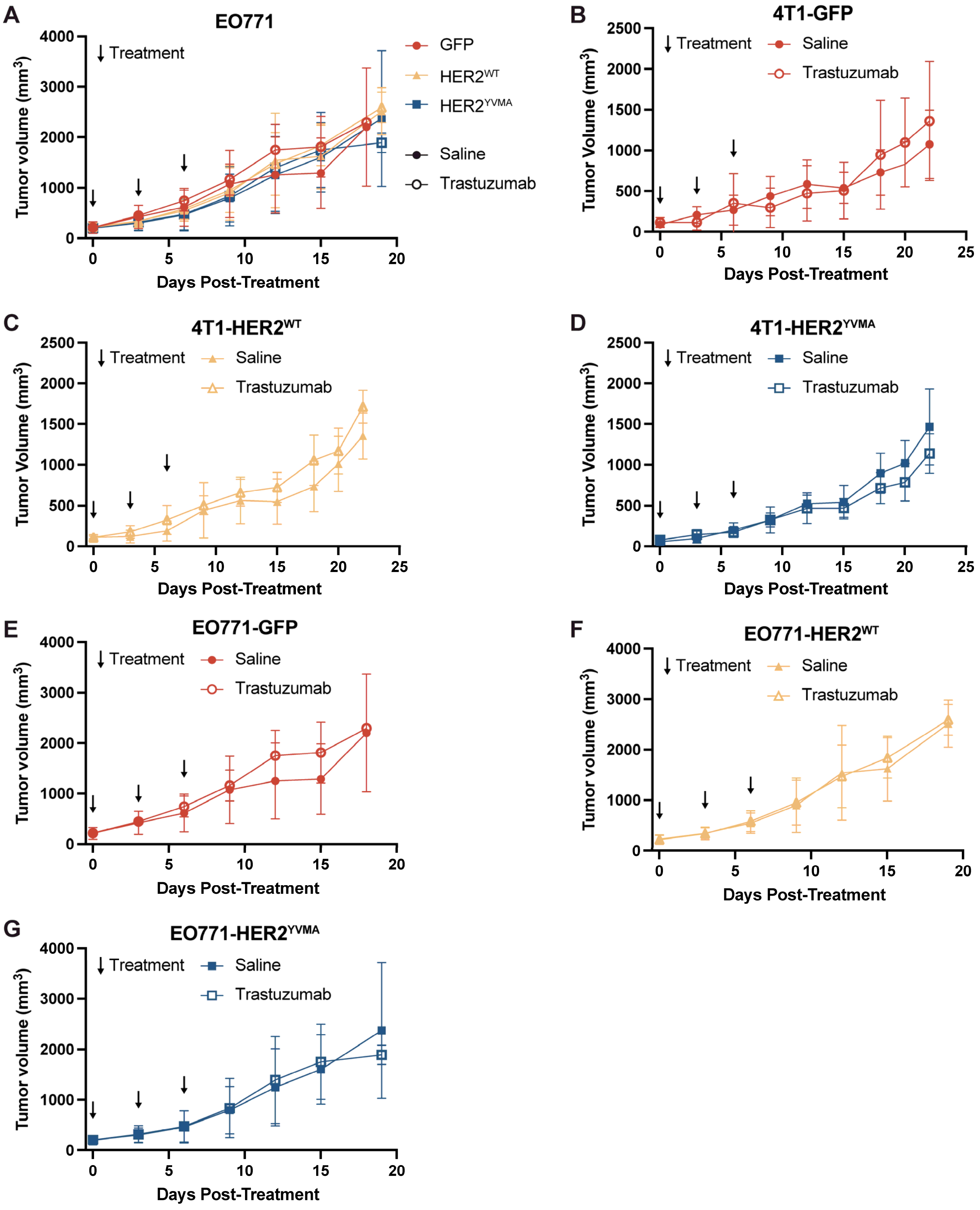

Supplemental Figure S3. Trastuzumab alone did not reduce primary tumor size in EO771-HER2 models. (**A**) EO771-GFP, EO771-HER2^{WT}, and EO771-HER2^{YVMA} tumors were treated with either saline or trastuzumab (15 mg/kg, IV). No significant difference in primary tumor volumes was found between different groups. No brain metastasis was observed at the end of the experiment. Individual tumor volume curves for (**B**) 4T1-GFP, (**C**) 4T1-HER2^{WT}, (**D**) 4T1-HER2^{YVMA}, (**E**) EO771-GFP, (**F**) EO771-HER2^{WT}, and (**G**) EO771-HER2^{YVMA}.

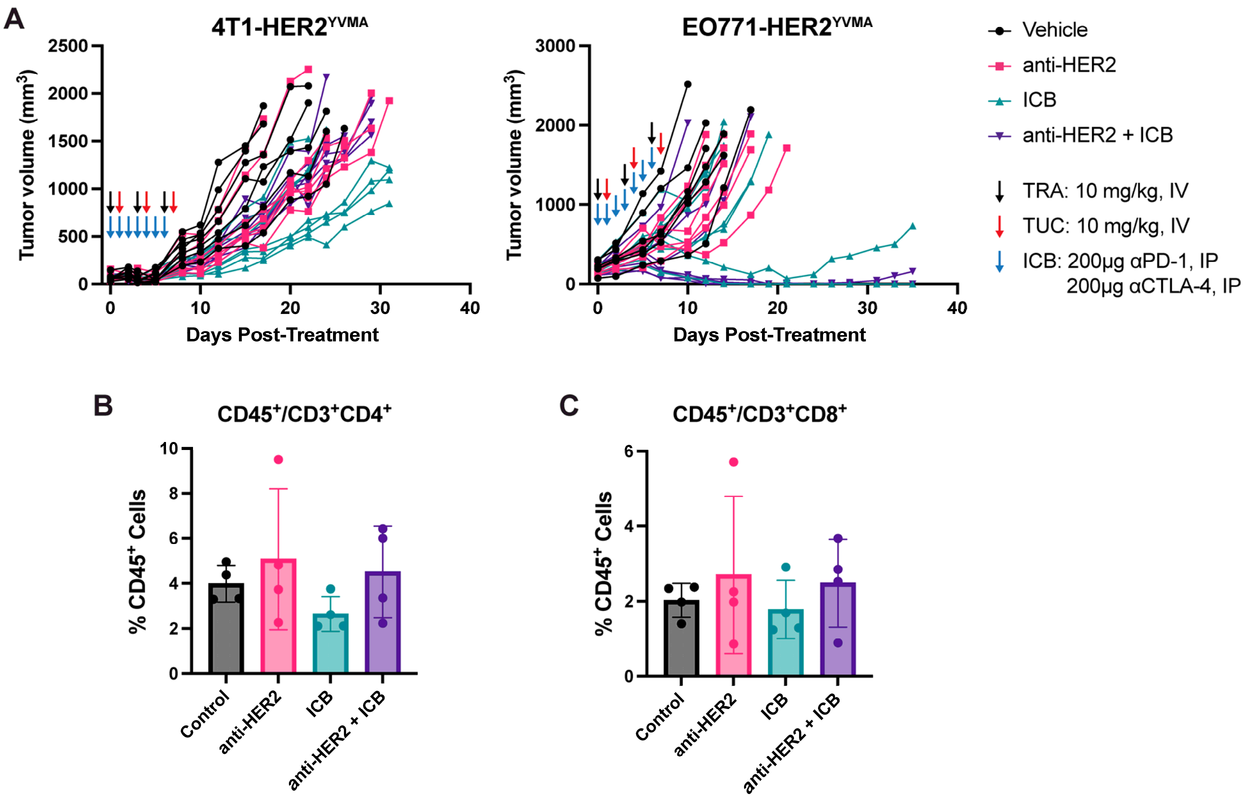

Supplemental Figure S4. HER2-targeted therapy and ICB combined in HER2-OE models. (**A**) Tumor growth curves for individual mice are shown for 4T1-HER2^{YVMA} (left) and EO771-HER2^{YVMA} (right) models treated with saline, anti-HER2 agents (TRA + TUC), or combination therapy (anti-HER2 + ICB). (**B**) Flow cytometry quantification of CD4+ (**B**) and CD8+ (**C**) T cells in 4T1-HER2^{YVMA} tumors on day 5. Two-way ANOVA with Bonferroni correction for multiple comparisons was used for (**B-C**). All data are mean ± SD; *p < 0.05; **, p<0.01.

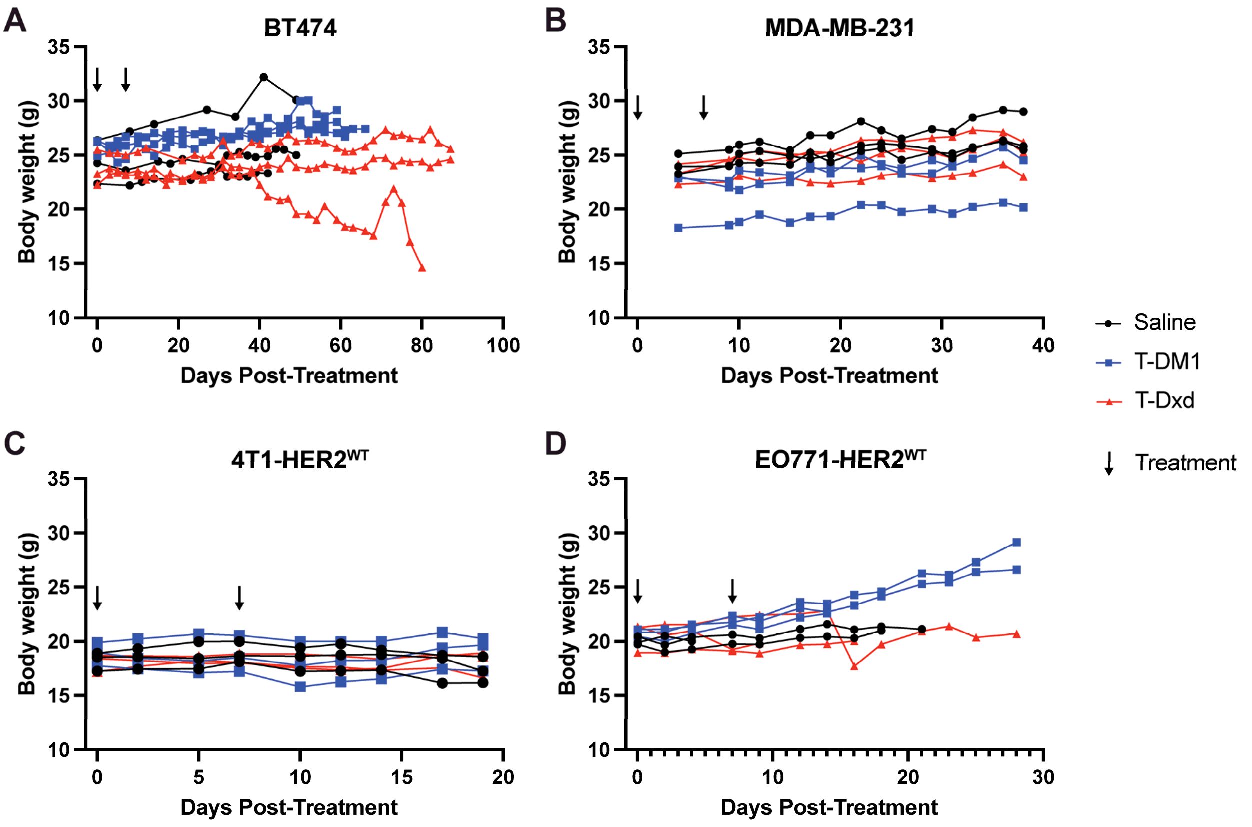

Supplemental Figure S5. T-DM1 and T-Dxd are well tolerated in breast cancer models. Mice body weight in (**A**) BT474 (human HER2+), (**B**) HER2– MDA-MB-231 (human TNBC), (**C**) 4T1-HER2^{WT}, and (**D**) EO771-HER2^{WT} treated with saline, T-DM1, or T-Dxd. N=3 per group.
