# Supplementary material for: Characterization of HER2-Positive Murine Breast Cancer Models for Investigating HER2-Targeted Therapy and Immunotherapy
Source: Cancers (Basel). 2026 Mar 19;18(6):997. doi: 10.3390/cancers18060997 (PMC13024896; doi:10.3390/cancers18060997)
Supplement: Supplementary file 1 [file cancers-18-00997-s001.zip › supp_file_S1.pdf]

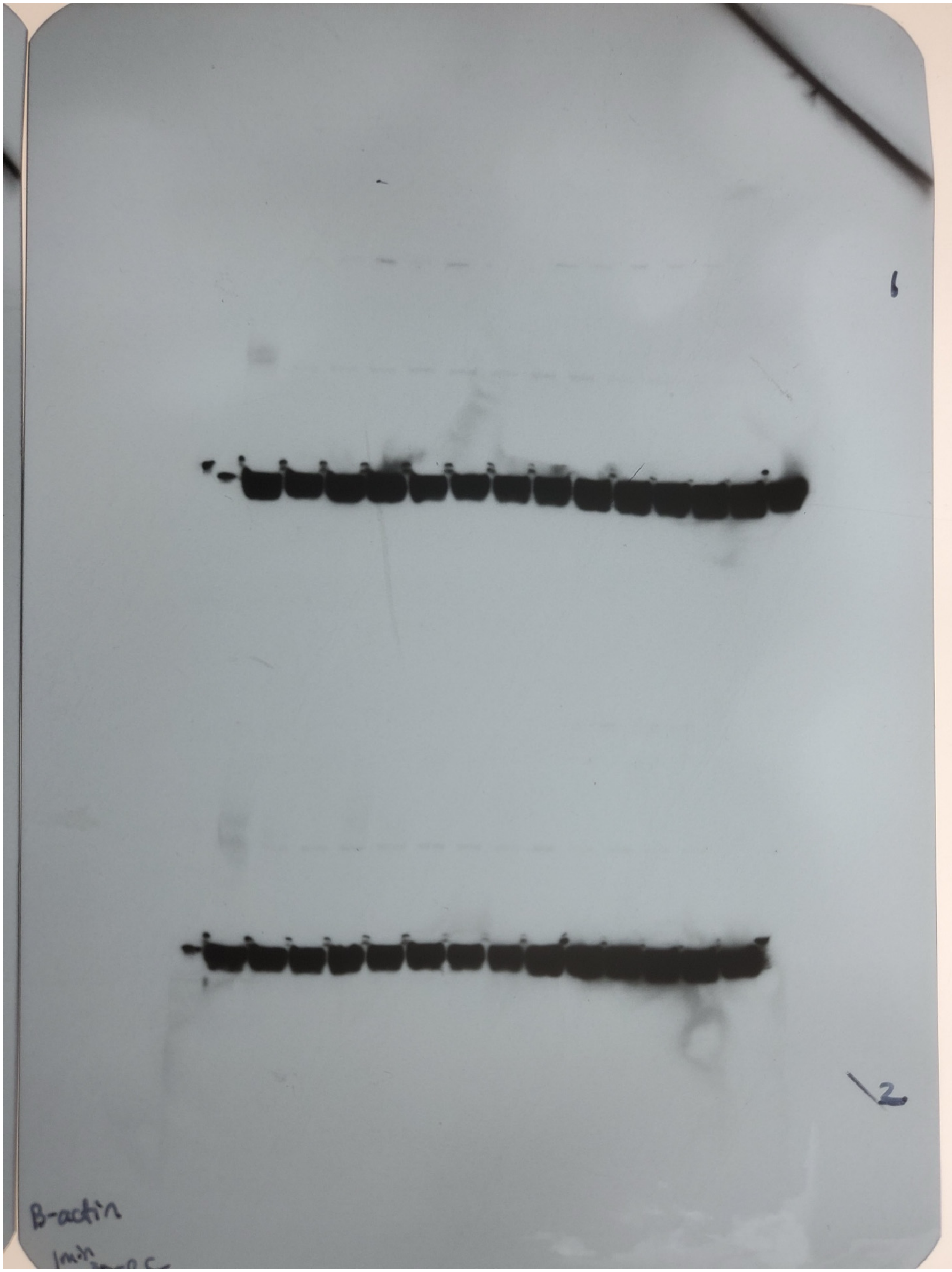

4T1 western blot stained for β-actin

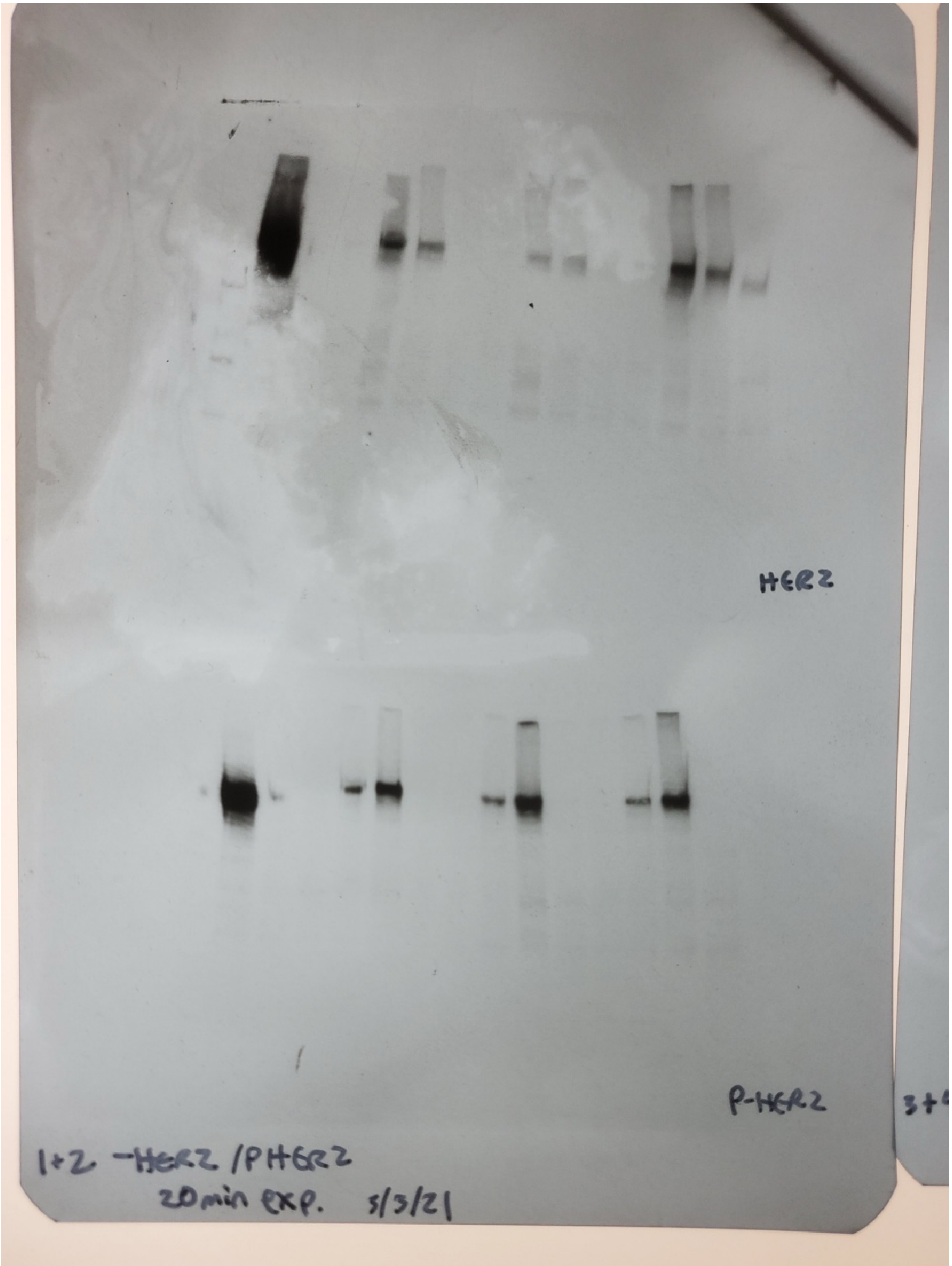

4T1 western blot stained for HER2 (top) and pHER2 (bottom)

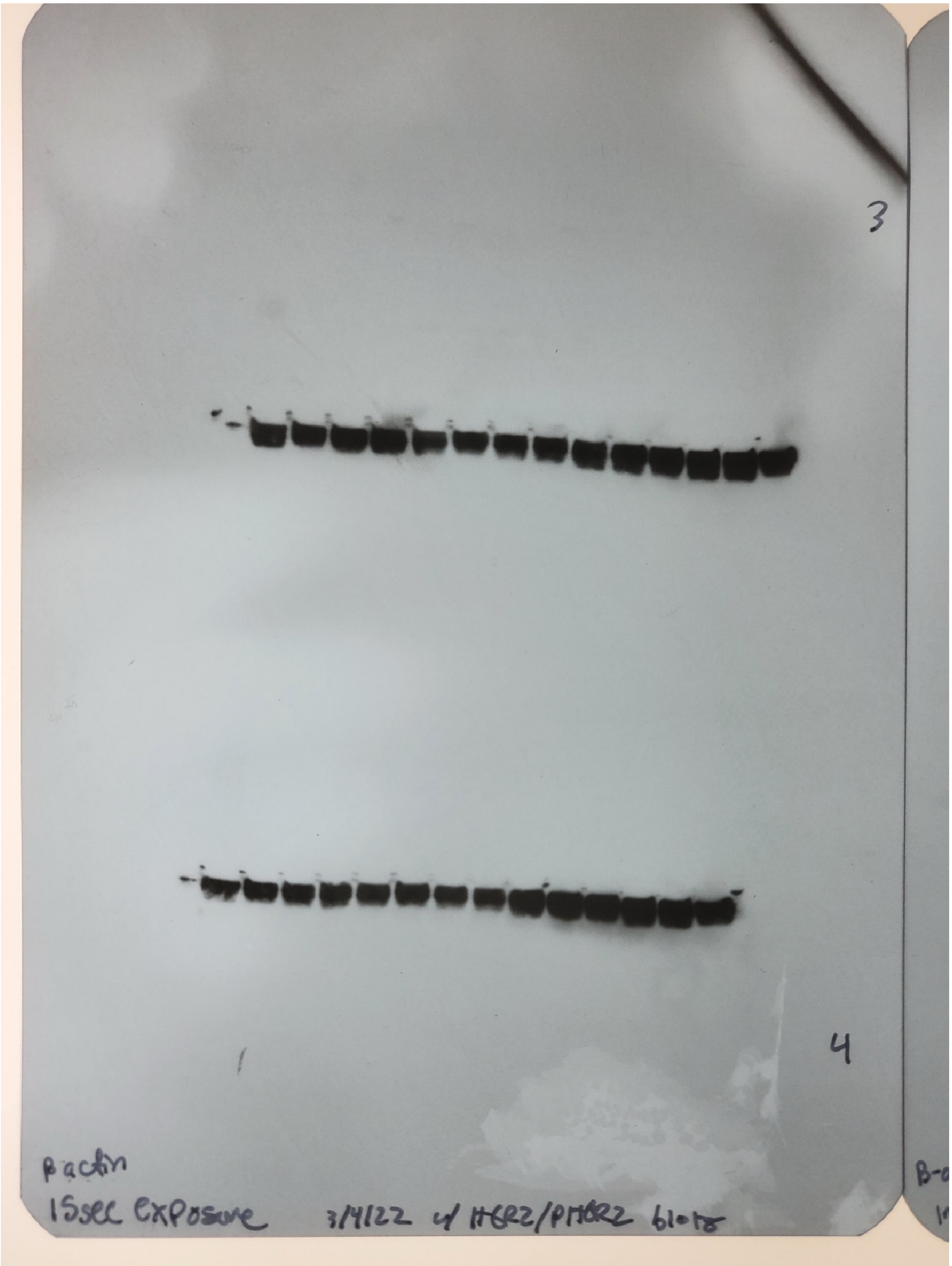

EO771 western blot stained for β-actin

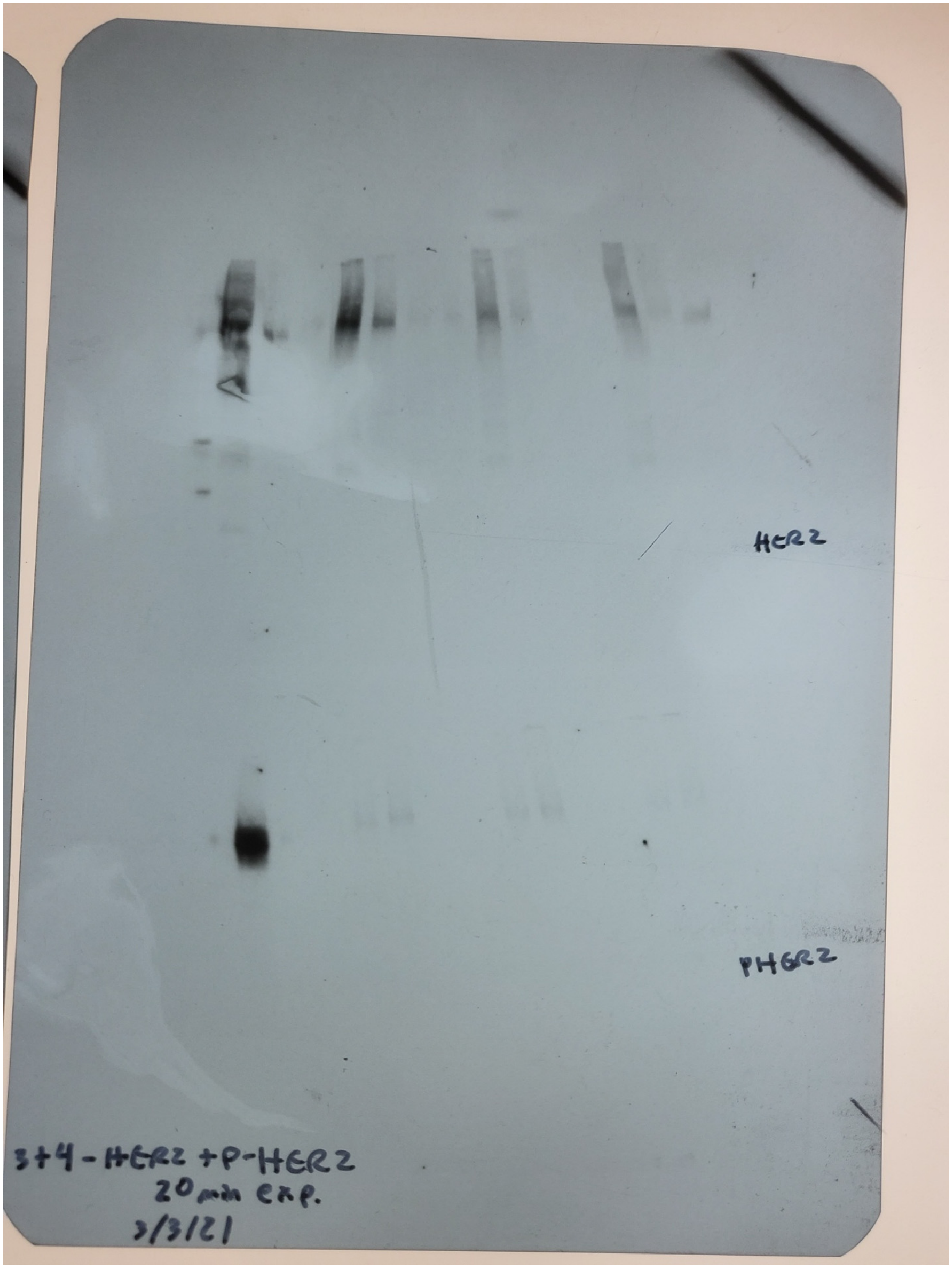

EO771 western blot stained for HER2 (top) and pHER2 (bottom)
